# The in vitro effect of electronic versus conventional cigarettes on color stability and surface roughness of dental resin composites

**DOI:** 10.1038/s41598-025-28238-5

**Published:** 2025-12-01

**Authors:** Ali Nadm Hmood, Maha Mohamed Ahmed Ebaya, Abeer El-Sayed El-Embaby

**Affiliations:** Department of Conservative Dentistry, Faculty of Dentistry, Mansoura University, Mansoura, Egypt

**Keywords:** Color stability, Conventional cigarettes, Dental restoration, Electronic cigarettes, Resin based composites, Surface roughness, Smoking, Health care, Materials science, Medical research

## Abstract

The appearance quality of dental restoration is recognized as crucial for the oral health-related quality of life. This study aimed to evaluate and compare the effect of electronic and conventional cigarettes on the color stability and surface roughness of dental composites. Eighty-four specimens of four composites (multi-shade: Tetric Prime, Neo Spectra ST-HV, and single-shade: Charisma Diamond One, Omnichroma; *n* = 21/material) were exposed to electronic cigarettes, conventional cigarettes, or artificial saliva (control) for 60 days using an automated smoking simulator with special robotic system for brushing, finishing, and polishing, mimicked six months of heavy smoking. Color and roughness were measured at baseline, after exposure, and post-thermocycling using a spectrophotometer and optical profilometer. Statistical analysis (SPSS v25) employed a three-way ANOVA, Bonferroni post hoc, and Pearson correlation (*p* < 0.05). The color stability was significantly different according to the type of treatment. In comparison, surface roughness was not affected by any treatment. The correlation between color stability and surface roughness was significant negative (p-value = 0.017). This study showed that conventional cigarettes caused higher discoloration than electronic cigarettes. While multi-shade resin-based composite materials had lower color stability than single-shade counterparts. On the other hand, neither smoking nor thermocycling negatively affected the surface roughness of materials.

## Introduction

Dental restoration refers to the procedure of placing a specialized material into a prepared cavity or damaged area of a tooth to preserve or restore its natural functions. These functions include the tooth’s mechanical ability to withstand the forces of chewing, its physiological role in maintaining oral health, and its anatomical structure, which supports the overall function and esthetics of the mouth. Additionally, it helps to prevent further decay of the tooth^1^. In today’s esthetically conscious society, the appearance quality of dental restoration is recognized as crucial for the oral health-related quality of life. Resin-based composites (RBCs) restorations have gained prominence due to their ability to be color-matched to the natural tooth, achieving the patient’s demand for an esthetically pleasing dental restoration, and it is matched with modern dental care, which emphasizes prolonging the tooth restoration cycle through minimally invasive procedures, aiming to preserve tooth tissue. However, these restorations are still susceptible to challenges such as staining, discoloration, and fractures^2,3^.

Advances in tooth-colored restorative materials have made it possible to closely mimic the natural appearance of teeth. This is achieved through blending or chameleon effects, enabling the restorative material to integrate successfully into the surrounding tooth structure. RBCs with enhanced optical properties and a variety of shades have been developed based on these demands. Indeed, single-shade RBCs designed with blending effects simplify the color-matching process for dentists by making treatments more efficient and patient-centered^4,5^. Patients are often concerned about the durability and quality of esthetic restorations, which are influenced by factors such as hygiene, diet, and smoking habits^6^. Conventional cigarettes (c-cigarettes) smoke, containing harmful substances like carbon monoxide and tar, can cause discoloration in teeth and dental restorations, affecting surface roughness, microhardness, water sorption, solubility, and staining susceptibility, emphasizing the need for improved dental care^7^.

A growing number of smokers have shifted to electronic cigarettes (e-cigarettes), battery-operated devices that resemble cigarettes or cigars and turn a solution of nicotine flavorings and other compounds into an aerosol that may be inhaled. These “modified risk tobacco products” contain fewer dangerous compounds than normal conventional cigarettes. Thus, they were offered as an alternative to traditional cigarettes and an intermediate stage in quitting smoking^8,9^. Surface roughness plays a significant role in the discoloration of dental restorations by trapping plaque and bacteria, as well as staining substances coming from different sources, such as food, beverages, and tobacco smoking. These trapped elements often result in noticeable color alteration after some time. Its degree is highly dependent upon the type of RBCs used and the quality of the finishing and polishing systems executed. Proper finishing and polishing are important factors in maximizing both the esthetics and durability of dental restorations and, thus, are mandatory clinical restorative procedures^10,11^.

Several studies have explored the color stability of RBCs restorations, employing artificial staining processes with liquid staining solutions. However, limited research has delved into the impact of smoking on the color stability and surface roughness of RBCs. Some investigations suggest that e-cigarettes have a similar discoloration impact on RBCs as c-cigarettes^12^. Conversely, other studies indicate that e-cigarettes cause less discoloration than c-cigarettes^13^. On the other hand, regarding the surface roughness of RBCs, some studies suggest alterations with smoking exposure^14^, while others contend that smoking has no discernible effect on surface roughness^15^. Given the advancements in RBC technology and the increasing use of both c-cigarettes and e-cigarettes, this research aims to evaluate their impact on the color stability and surface roughness of RBCs. By addressing this gap, the study seeks to provide valuable insights into the durability and esthetic performance of contemporary restorative materials in the context of smoking habits. The null hypotheses of this study were no significant difference of electronic cigarettes in comparison to conventional cigarettes on (first) color stability and (second) surface roughness of different composite resin materials.

## Materials

There were four types of RBCs used in the experiment: two multi-shade RBCs (Tetric Prime and Neo Spectra ST-HV), and two single-shade RBCs (Charisma Diamond One and Omnichroma). On the other hand, two tobacco exposure products: (JUUL) as e-cigarettes and (Marlboro Red), which was c-cigarettes (Table 1 for specifications). And other materials: Enhance Finishing system with Enhance PoGo Polishing system (Dentsply/Caulk, Milford, DE, USA), Pronamel Daily Protection toothpaste (Sensodyne ProNamel, GlaxoSmithKline, Turkey). Also, artificial saliva solution formulated by Mansoura University’s Department of Pharmaceutics (composition g/L: 23 g NaH₂PO₄, 11.8 g NaCl, 11.8 g KCl, 29.5 g urea; pH 6.8 ± 0.2)^16,17^.

**Table 1:** Materials used in this Study.

Material	Manufacturer	Classification	Composition	Batch Number
Tetric Prime (A2 shade)	Ivoclar Vivadent, Schaan, Liechtenstein	Nanohybrid	Matrix: Bis-GMA, UDMA, Bis-EMA. Filler (79–80 wt%): barium glass, YbF_3_, mixed oxide (SiO_2_-ZrO_2_), and copolymers. Fillers size ranges between 0.11 μm to 15.5 μm. Photoinitiator system: CQ, EDMAB, TPO, and Ivocerin.	Z062JX
Neo Spectra ST-HV (A2 shade)	Dentsply, Konstanz, Germany	Nanohybrid	Matrix: Urethane-modified Bis-GMA, TEGDMA, Bis-EMA. Filler (78–80 wt%): pre-polymerized fillers, barium glass, and YbF_3_. Fillers size 0.1 to 3.0 μm. Photoinitiator system: CQ, BPI, EDMAB.	2,311,000,229
Charisma Diamond One (single shade)	Kulzer GmbH, Hanau, Germany	Nanohybrid	Matrix: TCD-DI-HEA, UDMA, TEGDMA. Filler (81 wt%): B_2_O_3_-F-Al_2_O_3_-SiO_2_. Fillers size range between 5 nm to 20 μm. Photoinitiator system: CQ and EDMAB.	M010025
Omnichroma (single shade)	Tokuyama Dental, Tokyo, Japan	Nanofill	Matrix: UDMA, TEGDMA. Filler (79 wt%): SiO_2_-ZrO_2_. Fillers size 260 nm. Photoinitiator system: CQ and tertiary amine.	2886
Juul (electronic cigarette)	Juul Labs, San Francisco, USA	4th generation of electronic cigarette	Juul device rechargeable Juul pods are prefilled disposable cartridges (cartridges: 5% nicotine, Virginia tobacco flavor) containing nicotine 55.0 (mg/ml), PG 365.0 (mg/ml), VG 710.5 (mg/ml), PG/VG ratio 32/63.	ME27SA06A
Marlboro Red (conventional cigarette)	Philip Morris, Neuchâtel, Switzerland	Conventional cigarette	Tar 10 mg, nicotine 0.8 mg, carbon monoxide 10 mg, tobacco, water, sugars, PG, glycerol, licorice extract, diammonium phosphate, ammonium hydroxide, cocoa, cocoa products, carob bean and extract, natural and artificial flavors.	CN34233002

### Methods

The study protocol was approved by the Ethical Institution Committee of the Faculty of Dentistry at Mansoura University, Egypt, with approval number A0401024CD.

### Sample size calculation

The sample size was calculated based on the differential effects of cigarette smoking on the color change of dental resin-based composites, as reported in previous research^15^. A priori power analysis was performed using G*Power software (version 3.1.9.6, Düsseldorf, Germany) for a two-tailed independent t-test comparing two means. The analysis assumed an effect size (d = 2.23), a significance level of α = 0.05, and a target power of 95% (1 − β = 0.95). The calculation indicated that a minimum of seven specimens per subgroup was required, which was achieved in this study.

### Study design

The study used eighty-four specimens, which were assigned into four main groups based on the type of material (*n* = 21/group):

- Group T: Tetric Prime (Ivoclar Vivadent).
- Group N: Neo Spectra ST-HV (Dentsply).
- Group C: Charisma Diamond One (Kulzer GmbH).
- Group O: Omnichroma (Tokuyama Dental).

Then, by using a randomization through a standardized allocation concealment protocol using sequentially numbered, opaque, sealed envelopes^18^. Each material group was further divided into three exposure subgroups (*n* = 7/subgroup):

- Subgroups I (TI, NI, CI, OI): e-cigarette exposure (JUUL).
- Subgroups II (TII, NII, CII, OII): c-cigarette exposure (Marlboro Red).
- Subgroups III (TIII, NIII, CIII, OIII): control (artificial saliva immersion).

### Specimen preparation

A custom Teflon mold was designed to produce standardized disc-shaped specimens measuring 10 mm in diameter and 2 mm in thickness. A transparent celluloid strip (PD, Switzerland) with dimensions of 0.05 mm (thickness), 10 mm (width), and 20 mm (length) was positioned on an 8 mm thick glass slab (8 × 8 cm). The mold was then placed atop the strip, and the restorative material was applied in a single 2 mm increment using the bulk-fill technique, following the manufacturer’s instructions. To ensure proper adaptation and minimize air entrapment, a composite modeling instrument (CompoRoller, Kerr, Switzerland) was used to press the material into the mold cavity.

Following overfilling of the mold, the restorative material surface was covered with an additional celluloid strip and a second glass slab to prevent contamination. A standardized pressure of 500 g was applied for 20 s to ensure consistent stress distribution across all specimens^19^. Excess material was subsequently trimmed, and a fresh strip was used to smooth the surface, thereby reducing the formation of an air-inhibited layer^20^. Polymerization was performed using a light-emitting diode (LED) curing unit (Radii Xpert, SDI Limited, Australia) with an output intensity of 1500 mW/cm², as verified by the device’s integrated radiometer. Following the manufacturer’s instructions, each specimen was exposed to radiation for 20 s.

After polymerization, the specimens were carefully taken out of the mold and kept in distilled water (Integrative for Lab. Industries, Egypt, pH ≈ 7) in a dark incubator (DS20, BioStep, Egypt) at 37 ± 1 °C for 24 h to replicate the oral cavity environment, allowing for post-polymerization and the elution of unreacted components^21^. Finally, the specimens underwent a delayed finishing and polishing process^22^.

**Fig. 1:**
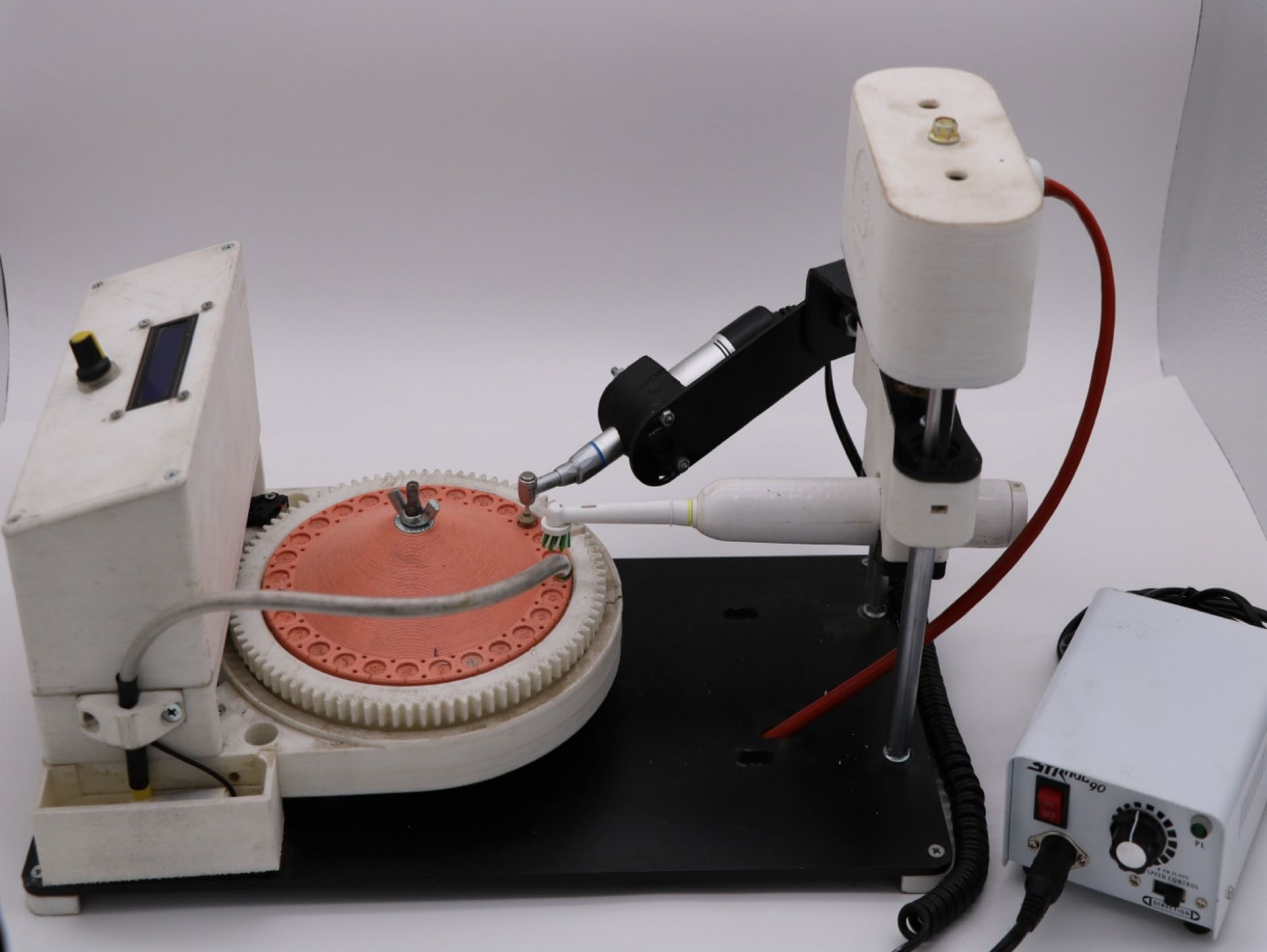
Custom-made brushing, finishing, and polishing robot.

To standardize the finishing and polishing procedures, ensure that the contact pressure force was two newtons and the contact time was two seconds^23^. They were applied on the surface of the specimen by a low-speed handpiece (NSK, Tokyo, Japan) for both finishing and polishing procedures to each specimen without water spray^24^. This was achieved by using a custom-made brushing, finishing, and polishing robot, which was connected to the Enhance Finishing System and Enhance PoGo Polishing System (Dentsply/Caulk, Milford, DE, USA) to reduce granulation by the low-speed handpiece that linked by a micro-motor (Strong 90-108E, Saeshin, Daegu, Korea) with preset speed at 20,000 rotations per minute (RPM) to decrease the surface roughness as much as possible and to remove the resin-rich layer (Fig. 1)^25,26^.

### Custom made Brushing, Finishing, and Polishing robot

The custom-made robot was used for brushing, finishing, and polishing procedures in this study (Fig. 1). It had an autorotating base into which the specimens holder was fitted (the specimens holder was constructed for twenty-eight specimens to be placed). Additionally, it included a nozzle that sprayed distilled water onto the specimens (for the brushing process), an automated toothbrush holder, a handpiece holder that linked with the micro-motor, and a control unit used to regulate the position, pressure, time of the toothbrush or the handpiece holder, water from the nozzle, and the rotation of the base from one specimen to another by special sensors. When using the machine in the brushing process, the holder arm of the handpiece is lifted to perform the brushing procedure.

### Study procedure

Following specimen preparation, baseline measurements (M1) were recorded for both color and surface roughness. The specimens were then subjected to a 60-day staining protocol using a custom-designed smoking simulation apparatus, developed to replicate the effects of heavy smoking over a six-month period^16^.

The smoking cycle was also automated to mimic the puffing regimen of a typical smoker. A 2-second inhalation and a concurrent 2-second air draw were incorporated in every cycle, with a 1-second transition time to allow for solenoid valve of the device switching between them. This was followed by a 15-second spraying interval of temperature-controlled artificial saliva (37 ± 1 °C) into the chamber that allowed specimen hydration and oral environment simulation. The whole cycle lasted 30 s and was delivered uninterrupted during exposure. This gave a reproducible and clinically relevant smoking behavior^16^.

The exposure conditions were categorized as follows:

- Subgroup I: Exposed to three electronic cigarette cartridges per day for 60 days.
- Subgroup II: Exposed to 60 cigarettes per day for 60 days.
- Subgroup III: Immersed in artificial saliva (control group, simulating non-smoker conditions).

Each experimental day was design to represent three days of heavy smokers life^27,28^. For c-cigarette exposure, smoke delivery was terminated 10 mm before the filter to standardize smoke concentration. Electronic cigarette devices were fully charged prior to each use. Specimens were systematically labeled on their base surfaces to indicate material type, exposure subgroup, and positional identifier within the holder of the robot. Also to prevent surface damage, specimens were handled carefully by their edges using laboratory tweezers. Artificial saliva was refrigerated (4–8 °C) to preserve its chemical stability and replaced every 48 h to mitigate microbial proliferation. Containers were sealed to minimize evaporation during incubation, and prior to each replacement, the saliva was equilibrated to room temperature^29^.

To mimic good oral hygiene, specimens were brushed once daily using an Oral-B PRO 1000 electric toothbrush (Procter & Gamble Service GmbH, Germany) fitted with a CrossAction brush head that attached to an automated robotic system. The brushing cycle lasted 3 s for each specimen, and with a steady flow of distilled water during each cycle. Before brushing, the brush head was moistened, and 0.4 g of Pronamel Daily Protection toothpaste (Sensodyne ProNamel, GlaxoSmithKline, Turkey) was applied. Throughout the procedure, a controlled contact force of 2 N was used^30^. After brushing, the samples were rinsed with distilled water for one minute^31^, and then put in an incubator with artificial saliva at 37 ± 1 °C for the whole 60-day exposure period.

After the staining procedure, the specimens were reevaluated for their color and surface roughness (M2). Following this, the specimens were aged using a thermocycling machine. Subsequently, the specimens were reevaluated using the same tests post-thermocycling (M3).

### Color stability testing (DE2000 or ΔE₀₀)

The color parameters of all specimens were measured at three time points: baseline (M1), after exposure to the staining agent (M2), and following thermocycling (M3). These measurements were performed using a UV-Vis-NIR spectrophotometer (Cary 5000, Agilent Technologies, Santa Clara, CA, USA). Color differences (ΔE00, or DE2000) were calculated between M2-M1 (effect of smoking), M3-M2 (effect of thermocycling), and M3-M1 (effect of smoking with thermocycling), using International Commission on Illumination CIEDE2000 (DE2000 or ∆E00) formula, based on the CIE L*a*b* color space system, with CIE standard illuminant D65 and a black background as references^32^. The ΔE00 formula is given by:

$$\:{\varDelta\:\text{E}}_{00}=\sqrt{{\left(\frac{\varDelta\:\text{L}{\prime\:}}{{K}_{L}{S}_{L}}\right)}^{2}+\:{\left(\frac{\varDelta\:\text{C}{\prime\:}}{{K}_{C}{S}_{C}}\right)}^{2}+{\left(\frac{\varDelta\:\text{H}{\prime\:}}{{K}_{H}{S}_{H}}\right)}^{2}+{\text{R}}_{T}\left(\frac{\varDelta\:\text{C}{\prime\:}}{{K}_{C}{S}_{C}}\right)\left(\frac{\varDelta\:\text{H}{\prime\:}}{{K}_{F}{S}_{H}}\right)\:}$$

Where ∆L was the lightness difference, ∆C was the chroma difference, ∆H was the hue difference, R_T_ was the interaction term for chroma and hue differences, and S_L_, S_C_, S_H_, K_L_, K_C_, and K_H_ constant coefficients. Spectral measurements were conducted across wavelengths from 380 to 780 nm at 1 nm intervals. The ΔE00 values were calculated using an Excel-based implementation of the CIEDE2000 formula^32^. These values were then compared to the perceptibility threshold, which represents the initial visually detectable color variation (∆E_00_ = 0.8), and the acceptability threshold, which represents the starting point of unacceptable color change (∆E_00_ = 1.8)^33^.

### Surface roughness evaluation (Ra)

The assessment of surface roughness (Ra) was performed for all samples at three separate time periods: baseline (M1), subsequent to exposure (M2), and after thermocycling aging (M3). A noncontact three-dimensional (3D) optical profilometer (Wyko, Model NT 1100, Veeco, Tucson, USA) was employed to assess the surface roughness. The profilometer was linked to a PC with image software (Vision 32, Veeco, USA)^17^.

The program utilized for generating the images provided mathematical roughness mean (Ra) data by analyzing the peaks and valleys in the specified area using a profilometer with a 0.8 mm cut-off and a 2.4 mm assessment length. Consequently, a 3D representation of the surface profile of the specimen was generated. Subsequently, a total of three pictures (3D) were collected for each specimen, namely in both the central and side regions, measuring 10 μm × 10 μm.

### Thermocycling

**Fig. 2:**
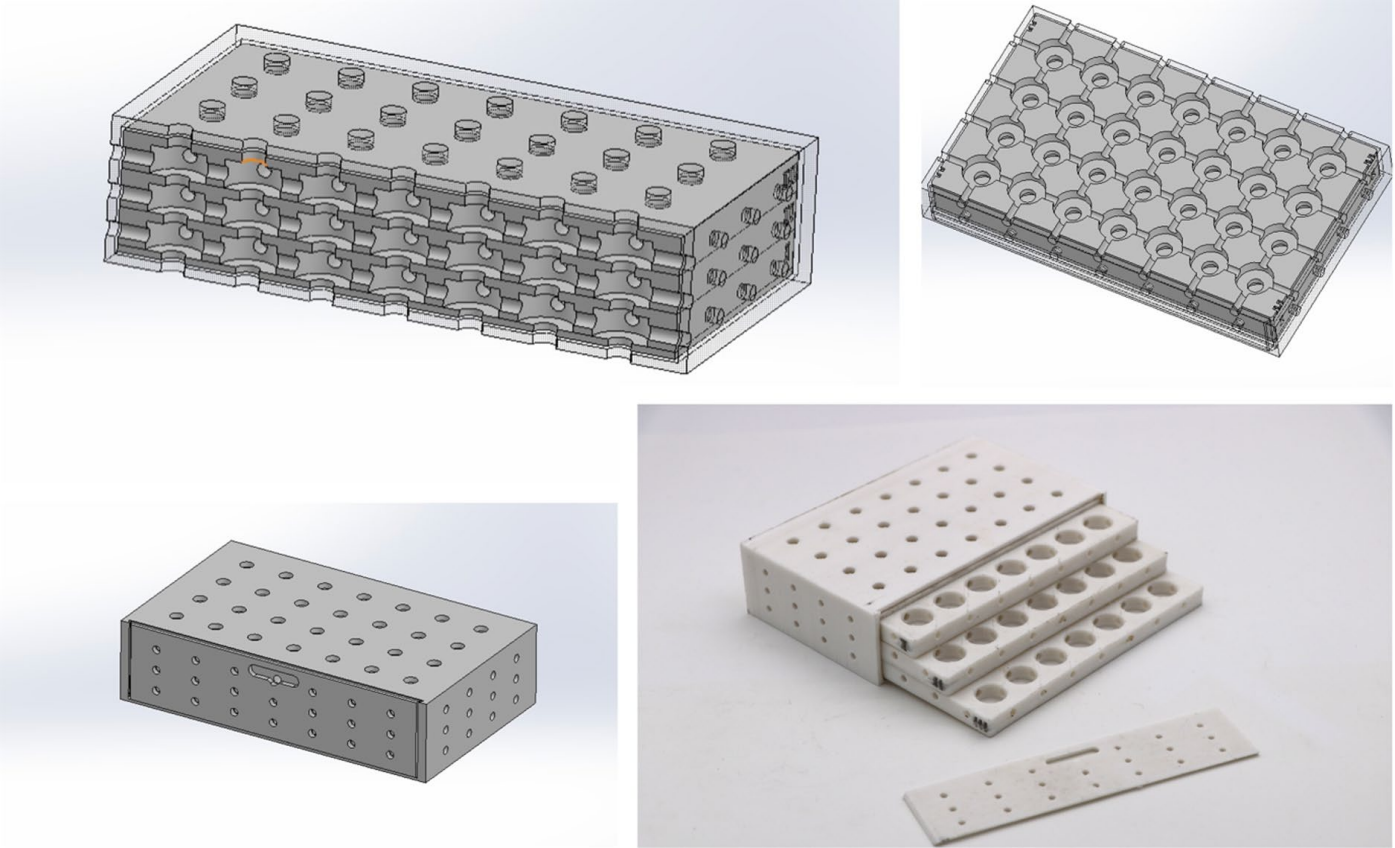
Custom specimen chamber for thermocycling.

After the second evaluation, all of the specimens were subjected to thermocycling, which was performed for 5000 cycles that simulated six months of clinical environments^15,34^. This was conducted using a thermocycling machine (Robota thermocycler, Alexandria, Egypt) consisting of two distilled water baths set at a median of 5 °C and 55 °C with dwell times of 30 s in each distilled water bath with a lag time of 10 s, as well as a custom specimen chamber for thermocycling (Fig. 2).

### Statistical analysis

The data were analyzed using the software SPSS (version 25, SPSS Inc., Chicago, IL, USA).

## Results

The Shapiro-Wilk tests were conducted for all variables, confirming that the data showed a parametric distribution. Descriptive statistics (means and standard deviations) were used to summarize the data. For comparisons of color change (DE2000 or ΔE₀₀) and surface roughness (Ra) between different groups (materials), subgroups (exposure), and treatments (time), a three-way repeated measures Analysis of Variance (ANOVA) was conducted, complemented by the Bonferroni test for multiple comparisons. DE2000 was also correlated with the color metrics DL, Da, and Db by using Pearson’s product-moment correlation coefficient, as well as DE2000 with Ra. The level of significance for all statistical tests was set at a p-value of < 0.05, and results were represented graphically by clustered bar charts to ensure clarity in interpreting the results.

### Color change (DE2000 or ΔE₀₀)

**Fig. 3:**
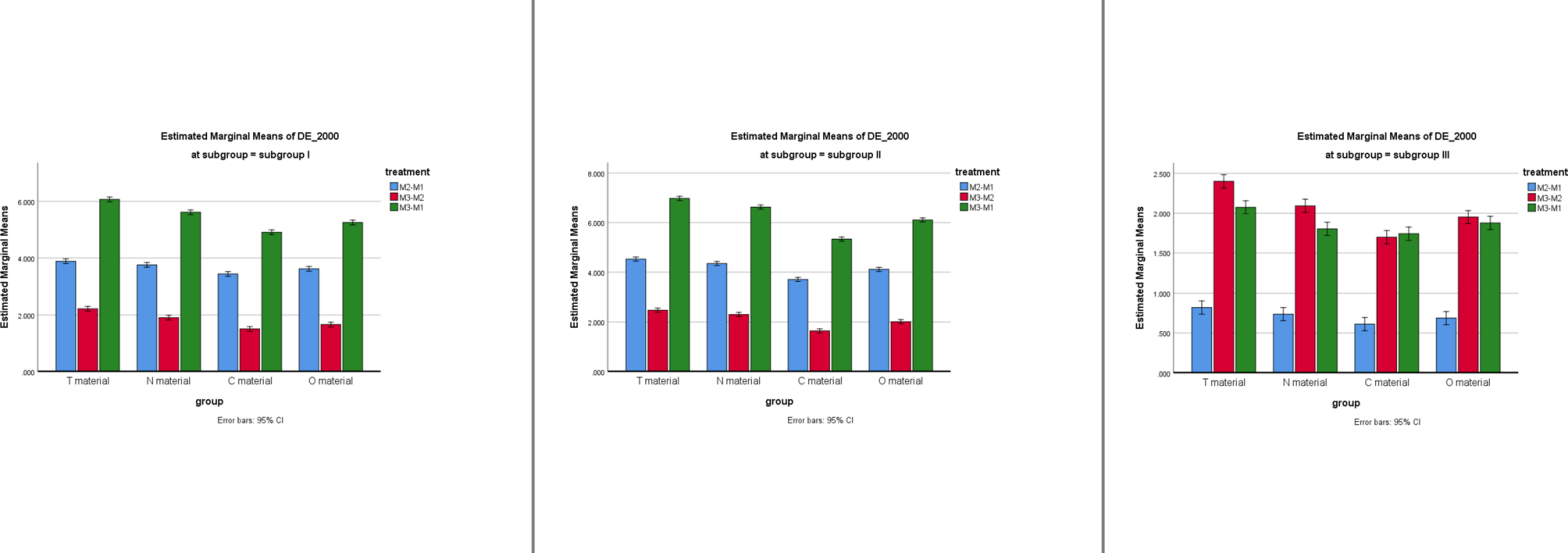
Comparison of DE2000 between treatments at different groups for subgroups.

ANOVA showed significant main effects of treatment, group, and subgroup (all *p* < 0.001), as well as significant two- and three-way interactions (Table 2; Fig. 3).

**Table 2:** Comparison of DE2000 between treatments for different groups and Subgroups.

Subgroups	Materials	M2-M1 (effect of exposure)		M3-M2 (effect of thermocycling)		M3-M1 (effect of both exposure with thermocycling)		Three-way ANOVA (*p* value)
		*X*	*SD*	*X*	*SD*	*X*	*SD*	
Subgroup I (e-cigarette)	T material	3.8814a	0.1167	2.2186b	0.0967	6.0700c	0.1800	< 0.001*
	N material	3.7543a	0.1175	1.9029b	0.1011	5.6143c	0.1609	< 0.001*
	C material	3.4357a	0.1028	1.5057b	0.1044	4.9086c	0.1423	< 0.001*
	O material	3.6143a	0.1078	1.6600b	0.0971	5.2529c	0.1294	< 0.001*
Subgroup II (c- cigarette)	T material	4.5271a	0.1216	2.4771b	0.1061	6.9771c	0.1390	< 0.001*
	N material	4.3471a	0.1098	2.3057b	0.0871	6.6271c	0.1608	< 0.001*
	C material	3.7014a	0.1004	1.6429b	0.0692	5.3343c	0.0796	< 0.001*
	O material	4.1114a	0.1132	2.0143b	0.1008	6.1086c	0.1849	< 0.001*
Subgroup III (control)	T material	.8200a	0.0702	2.4000b	0.1105	2.0757c	0.1320	< 0.001*
	N material	.7371a	0.0685	2.0942b	0.0799	1.8029c	0.0431	< 0.001*
	C material	.6114a	0.0696	1.6986b	0.0790	1.7414b	0.1122	< 0.001*
	O material	.6871a	0.0820	1.9514b	0.0919	1.8771b	0.0848	< 0.001*

X; mean of DE2000, SD; standard deviation; *p is significant at 5% level. Different lower-case letters in the same raw showed a significant difference between each 2 treatments (Bonferroni test, *p* < 0.05). Similar lower-case letters in the same raw showed non- significant difference.

### Treatment effect

Subgroup I: the highest values of DE2000 were found for treatments M3-M1 (effect of both exposure with thermocycling), followed by M2-M1 (effect of exposure), whereas the smallest color changes took place between treatments M3-M2 (effect of thermocycling).

Subgroup II: the DE2000 between M3-M1 (effect of both exposure with thermocycling) had the most obvious change, with a significant yet relatively less clear difference between M2-M1(effect of exposure), while the change between M3-M2 (effect of thermocycling) was the smallest.

Subgroup III: the highest DE2000 was shown between treatments M3-M1 (effect of both artificial saliva immersion with thermocycling), followed by M3-M2 (effect of thermocycling), whereas the lowest one was between treatments M2-M1 (effect of artificial saliva immersion).

- **Subgroup effect**: Subgroup II (c-cigarettes) produced the greatest discoloration, followed by Subgroup I (e-cigarettes), while Subgroup III (control) showed minimal change (*p* < 0.001).
- **Material effect**: Group T consistently showed the highest ΔE00 values, followed by N, O, and the lowest in C. In Subgroup III, only Group T differed significantly from the others.

### Correlation between DE2000, DL, Da, and Db

As shown in Table 3, presents the Pearson linear correlation among various color difference parameters: DE2000, DL, Da, and Db. It may be observed from this analysis that the relationship of DL with all other parameters is highly negative; an increase in DL provides a decrease in Da, Db, and DE2000 and vice versa. On the other hand, significant positive relationships may be established among Da, Db, and DE2000. An increase in Da is related to an increase in Db and DE2000, and vice versa: an increase in Db acts to increase DE2000.

**Table 3:** Correlation between DE2000, DL, Da, and Db.

Correlations					
		DL	Da	Db	DE2000
**DL**	**Pearson Correlation**	1	− 0.648^**^	− 0.950^**^	− 0.980^**^
	**Sig. (2-tailed) p-value**	-	< 0.001*	< 0.001*	< 0.001*
**Da**	**Pearson Correlation**	− 0.648^**^	1	0.659^**^	0.646^**^
	**Sig. (2-tailed) p-value**	< 0.001*	-	< 0.001*	< 0.001*
**Db**	**Pearson Correlation**	− 0.950^**^	0.659^**^	1	0.982^**^
	**Sig. (2-tailed) p-value**	< 0.001*	< 0.001*	-	< 0.001*
**DE2000**	**Pearson Correlation**	− 0.980^**^	0.646^**^	0.982^**^	1
	**Sig. (2-tailed) p-value**	< 0.001*	< 0.001*	< 0.001*	-

**. Correlation is significant at the 0.01 level (2-tailed).

### Surface roughness (Ra)

**Fig. 4:**
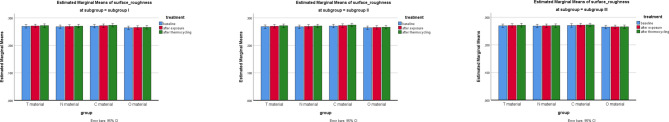
Comparison of Ra treatments at different groups for subgroups.

ANOVA revealed no significant effect of treatment or subgroup on Ra (*p* > 0.05). Only the group factor was significant (*p* < 0.001), with minor differences between materials. Across all conditions, changes in Ra remained clinically negligible (Table 4; Fig. 4).

**Table 4:** Comparison of Ra treatments for different groups and Subgroups.

Subgroups	Materials	M1 (Baseline)		M2 (After exposure)		M3 (After thermocycling)		Three-way ANOVA (*p* value)
		*X*	*SD*	*X*	*SD*	*X*	*SD*	
Subgroup I (e-cigarette)	T material	.2692a	0.0052	.2705a	0.0043	.2718a	0.0091	0.755
	N material	.2676a	0.0086	.2687a	0.0089	.2700a	0.0117	0.855
	C material	.2700a	0.0150	.2715a	0.0073	.2732a	0.0052	0.738
	O material	.2640a	0.0044	.2649a	0.0080	.2655a	0.0034	0.754
Subgroup II (c- cigarette)	T material	.2680a	0.0129	.2695a	0.0082	.2714a	0.0083	0.283
	N material	.2676a	0.0174	.2689a	0.0039	.2704a	0.0078	0.822
	C material	.2703a	0.0118	.2720a	0.0116	.2740a	0.0067	0.413
	O material	.2641a	0.0028	.2652a	0.0037	.2660a	0.0033	0.281
Subgroup III (control)	T material	.2690a	0.0057	.2701a	0.0058	.2708a	0.0056	0.332
	N material	.2681a	0.0113	.2691a	0.0032	.2696a	0.0045	0.320
	C material	.2701a	0.0107	.2713a	0.0099	.2722a	0.0089	0.833
	O material	.2644a	0.0025	.2652a	0.0066	.2655a	0.0065	0.351

X; mean of Ra, SD; standard deviation; *p is significant at 5% level. Different lower-case letters in the same raw showed a significant difference between each 2 treatments (Bonferroni test, *p* < 0.05). Similar lower-case letters in the same raw showed non- significant difference.

### Correlation between DE2000 and Ra

As revealed in Table 5, the data show a significant negative correlation between DE2000 and Ra, as the p-value is 0.017. This can be interpreted to mean that the higher the DE2000, the higher the color difference, which decreases the surface roughness and vice versa.

**Table 5:** Correlation between DE2000, and Ra.

Correlations			
		DE2000	Ra
**DE2000**	**Pearson Correlation**	1	− 0.150
	**Sig. (2-tailed)**		0.017*
**Ra**	**Pearson Correlation**	− 0.150	1
	**Sig. (2-tailed)**	0.017*	

**. Correlation is significant at the 0.01 level (2-tailed).

## Discussion

Improving the performance of dental materials requires studying their behavior and properties. While in vivo tests are important for understanding the physical properties, in vitro studies provide valuable information in a controlled environment^35^. Although the health hazards of smoking are well known, about 1.3 billion people around the world continue to use tobacco products, according to the World Health Organization. This widespread use is another reason for evaluating the impact of smoking on the esthetic and physical properties of dental materials^6^. Among the most extensively studied materials in dental schools, RBCs have been specially researched regarding their stability over long periods under oral conditions^36^.

Nanofilled and nanohybrid RBCs were selected in this study due to their widespread use in dental clinics for restorations in both anterior and posterior teeth, and they represent materials with superior physicochemical and mechanical properties^37^. Single-shade RBCs come in one universal shade created to match all 16 VITA Classic shade tabs. In contrast, multi-shade RBCs are offered in a limited range of shades, each one developed to match a specific set of shade Tabs^38^. Among these, the A2 shade is widely used and considered a standardized shade for RBCs specimens because it allows better evaluation of color changes, while its variation will have fewer influences^39^.

The investigation utilized that type of e-cigarettes due to their market dominance, standardized nicotine content, consistent vapor production, and chemical composition, making them representative of real-world usage. As such, they are the best candidates to perform studies on health impacts, material staining, and comparisons to c-cigarettes, ensuring relevant and reproducible findings^40^. Also, that type of c-cigarettes was included in this research due to its global popularity, standardized nicotine and tar content, and strong relevance in studying the health and material impacts of conventional smoking. It serves as a benchmark against which alternatives like e-cigarettes can be compared for staining effects, health risks, and addiction behaviors. It ensures findings that are reliable and applicable for smoking-related studies^41^.

The research uses a one-step finishing and polishing system for smooth, esthetically pleasing, and durable surfaces in studies involving RBCs. This system offers predictable results, resists discoloration and plaque accumulation, and provides consistent data, making it a benchmark tool in clinical practice and dental material research^42^. Also, the experiment utilized artificial saliva as a standardized medium for oral condition stimulation, allowing comprehensive studies on RBC properties, improving understanding, and developing durable dental restorations^43^. In addition, a low-abrasive relative dentin abrasion (RDA) toothpaste was employed in the present study due to its common use. Previous research concludes that abrasive toothpaste preserves the esthetic appearance and integrity of teeth and dental restorations^44^.

The standardization of finishing and polishing in the current research by using a custom-made brushing, finishing, and polishing robot with two N of contact pressure and two seconds of contact time provides consistent and reproducible results with a minimum surface roughness and without compromising the integrity of resin composites. This enhances esthetics, durability, and functionality; reduces operator variability; and is highly suitable for research settings. This decreases the surface roughness as much as possible and removes the resin-rich layer^25,26^.

The specimens were kept at 37 °C for 60 days to mimic a six-month clinical exposure period for heavy smokers. Each experimental day was equal to three clinical days, considering a 3:1 time compression ratio. Subgroups were given either three 5% nicotine e-cigarette cartridges (the same as one cartridge per day in actual life) or sixty cigarettes (the same as twenty cigarettes per day in real life) per day^27,28^. Control subgroups, on the other hand, only had access to artificial saliva. All specimens were concurrently incubated in artificial saliva at 37 °C to simulate the dual exposure to both tobacco byproducts and oral fluids characteristic of the intraoral environment.

To address a notable gap in prior research^45^, this study incorporated a tooth-brushing simulation, a critical factor often omitted in vitro but significant in reducing staining. Thus, this research used the custom-made brushing, finishing, and polishing robot to simulate good oral hygiene; each disc was brushed once daily for three seconds, with the contact force of the brush head on the specimens set to two newtons (2 N) with a flow of distilled water. This regimen represents the equivalent of twice-daily cleaning for a single tooth surface which equaled the calculated average brushing time per tooth surface under the assumption of a 2-minute overall brushing time for a complete dentition of 28 teeth and twice-daily brushing, as described by Lorenz et al.^30^ this conservative brushing regimen was selected to simulate daily oral hygiene with less abrasion that could potentially reduce staining effects.

Also, this study employed a custom-made smoking simulation machine with computerized smoking cycles that ideally replicate natural inhalation patterns. The system provided the same smoke/air distribution to all the specimens, resulting in similar exposure conditions and enhancing experimental consistency^7,16^. Quantitative color and surface measurements were taken at three critical time points: baseline (M1), after exposure (M2), and post-thermocycling (M3). This rigorous schedule allowed proper evaluation of material performance under uniform conditions of combined tobacco exposure and thermal cycling. Additionally, all experiments were performed by a single operator for methodological consistency.

Spectrophotometry with the CIEDE2000 (ΔE00) method is recommended in dentistry for better accuracy in assessing color differences, as it provides a closer match to visual evaluations compared to CIEDE1976 (ΔE*ab). This is due to ΔE00 considering lightness, chroma, hue, and their interactions; therefore, ongoing investigation used it. The resulting ∆E00 values were compared to two thresholds: the perceptibility threshold (ΔE₀₀= 0.8), which marks the smallest visible color change, and the acceptability threshold (ΔE₀₀= 1.8), which marks the point where the color change becomes unacceptable^33^.

A noncontact digital profilometer microscope, using a laser to create a 3D surface map, is preferred for surface evaluation as it is fast, non-damaging, and easy to use. So, the current study used that. The surface roughness of dental materials is usually measured in micrometers (µm), and a value of 0.2 μm is considered critical. A surface roughness higher than 0.2 μm is clinically significant because it enhances the possibility of biofilm accumulation, which could be one of the factors causing gingival inflammation and extrinsic discoloration. It may become particularly critical in affecting the esthetic and functional longevity of dental restorations, making control of surface roughness one of the most important aspects in the design and maintenance of dental materials^17^.

In this investigation, thermocycling has simulated six months of oral thermal changes, submitting specimens for 5000 cycles for 30 s in different temperatures ranging from 5 °C to 55 °C. A custom specimen chamber was designed with openings to ensure that the distilled water reached all the specimens equally, maintaining their material name, exposure group, and number. This process effectively emulates the expansion and contraction of dental materials due to changes in temperature resulting from food and liquid intake. It allows material durability, bond strength, color stability, and surface integrity during long thermal stress exposure to be duly assessed by providing insights into material longevity in an accelerated manner^34^.

Depending on the results of color stability in this study, the first null hypothesis was rejected. The color difference for the exposure effect (M2-M1) showed that c-cigarette smoking causes a higher grade of discoloration in restorations than the use of e-cigarettes, which also resulted in unacceptable discoloration. These results were in agreement with Alnasser HA et al.^46^, Paolone G et al.^13^, Abdulla R et al.^47^, and Hmood A et al.^16^, reinforcing c-cigarettes smoking as a primary etiological factor in dental staining. The observed discrepancy with Vohra F et al.^12^ conclusion of comparable discoloration between cigarette types may be attributed to methodological variations, including differences in smoking materials, exposure apparatus, measurement protocols, or experimental smoking regimens.

This noticeable discoloration might be mostly due to the tar component in c-cigarette smoke, which has strong adhesive and pigment properties, making it a major cause of staining with c-cigarette smoke. In addition to tar, burning tobacco creates other chromogenic compounds, such as caramelized sugars and additives like cocoa, which make the discoloration even worse. Burning tobacco can also change the way restorative materials appearance by thermal degradation. On the other hand, using an electronic cigarette (e-cigarette) can cause minor discoloration, but its effects are much less severe than those of a cigarette. This difference highlights the basic differences in the physicochemical properties of the two types of smoke, especially in how they stain and how they are made up^48^.

On the other hand, the findings of color difference for the thermocycling effect (M3-M2) agree with Naji W et al.^49^ who concluded that thermocycling significantly affects the color stability of RBCs. While disagreeing with Tuncer S et al.^50^ who reported that thermocycling did not influence the color stability of RBCs, this disagreement could be due to the different materials used in their study. Besides that, the findings of current research, when compared between (M2-M1) and (M3-M2) except control RBCs (subgroup III), showed that the exposure had an impact on the color of RBCs greater than thermocycling, and these outcomes agree with Hajj R et al.^51^ reported that thermocycling slightly affects the color stability of RBCs compared to exposure with colorants. These findings were also confirmed by El-Rashidy A et al.^52^

Furthermore, the current study noted that in M3-M2, subgroups II and III showed more discoloration than subgroups I; this might be due to the absorption of moisture in subgroups II and III, which can exacerbate material degradation during thermocycling. These factors increase susceptibility to discoloration during thermocycling, as Mathias P et al.^53^ reported that the c-cigarettes smoke significantly increased water sorption for RBCs, in which the smoke may alter the physical properties of RBCs. On the other hand, subgroups III was exposed for a longer time than other subgroups to artificial saliva, which was the major component of water that could enhance the effect of thermocycling. As Gusmão GM et al.^54^ concluded, water sorption of RBCs was dependent upon their storage time. According to Bociong K et al.^55^ water sorption impacts the physical properties of RBCs materials. As well as the study of Huang W et al.^56^ who found there was a positive correlation between water sorption and color change of RBCs materials. While the greatest color change for all subgroups of the present study was the effect of both exposure and thermocycling (M3-M1), and that was confirmed by Biçer, Z et al.^57^, and Koc-Vural U et al.^58^.

This color change could be due to organic constituents and ester linkages; RBCs make them hydrophilic in nature, which face moisture uptake while exposed to the oral environment. Often, water uptake leads to hygroscopic and hydrolytic effects that could eventually cause extrinsic staining, optical instability, and degradation of chemical bonding between filler and resin matrix. The water sorption value depended on the resin matrix^9^. Thermocycling can dramatically affect the structure and properties of resin materials, mostly via water absorption. Water absorbed from the external medium causes swelling of the polymer network of the resin, lowering friction between chains of the network, which negatively affects its mechanical strength and flexibility. Further, the presence of absorbed water inside the composite structure may hydrolyze the silane coupling agent, causing a deterioration of the chemical bond at the interface of the filler and resin matrix. Besides that, there is a thermal expansion coefficient mismatch between filler particles and the resin matrix. Internal stresses during temperature alterations may lead to crack formation or even partial separation of the fillers from the matrix^59,60^.

As shown in this study, groups T and N materials (multi-shade RBCs), respectively, are more susceptible to staining and had higher color changes (low color stability) compared to groups O and C materials (single-shade RBCs), respectively. That outcome is for both (M2-M1) and (M3-M2) color parameters. These results agree with Hmood A et al.^16^ reported that C RBCs had more color stability than N RBCs. But disagree with Alex A et al.^61^ who reported that multi-shade RBCs had more color stability than single-shade RBCs. Also, disagree with El-Rashidy A et al.^60^ and Khayat W.^62^ concluded that the color stability of single-shade RBCs is about the same as that of multi-shade RBCs. On the other hand, Alalawi, H.^63^ reported that T RBCs have more color stability than O RBCs, whilst disagreeing with Maghaireh, GA et al.^64^ who report N RBCs showed more color stability than C and O RBCs, and disagree with Duzyol, M et al.^65^ which reports that O RBCs have more color stability than C RBCs. These disagreements might be due to different staining exposure materials than the current study.

The different ways that dental composite resins can be stained are mostly due to differences in the resin matrix formulations and the properties of the filler particles. The resin matrix composition is the most important factor in how easily a material can change color^66^. TCD-urethane polymers, which were found in C RBCs have big molecules and no diluent monomers, which makes the colors of C RBCs more stable. These molecular features make the chemical structure more stable in two ways: the bulky TCD-urethane structure makes it harder for reactive sites to get to, and the lack of diluting agents removes any weak points in the polymer network that could lead to degradation pathways^67^.

The presence of UDMA-TCD (TCD-DI-HEA) copolymer formulations in C RBCS makes it more stable, compared to standard Bis-GMA-based composites in the T and N RBCs material groups, which exhibit significantly lower levels of residual double bonds and higher conversion rates. The TCD-DI-HEA matrix system, which only C RBCs have and O RBCs don’t, gives two major advantages: it prevents them from absorbing excessive amounts of water and makes them less thick, these characteristics improve mechanical performance and color stability. So, they are especially capable of withstanding the hard conditions found in the mouth^68^.

The intrinsic hydrophilicity of the Bis-GMA-based matrices in T and N RBCs, as opposed to UDMA and other methacrylate-based systems, leads to increased water sorption and, consequently, decreased stain resistance^69^. When TEGDMA concentrations in Bis-GMA resins are progressively raised from 0% to 1%, experimental data indicate that water uptake increases dependently from 3% to 6%, further exacerbating this effect^70^. It’s interesting to note that this compositional feature of N RBCs formulations increases their susceptibility to hydrolytic degradation and discoloration.

Also, the color of dental composites is highly influenced by characteristics such as the amount, size, shape, and type of fillers^71^. The RBCs examined in this study were about the same filler load, T (79–80 wt%), N (78–80 wt%), C (81 wt%), and O (79 wt%), so the higher filler load was in C RBCs groups followed by O RBCs groups, then T and N, which is aligned with accordance to color stability results. All tested materials were nanohybrid RBCs except the O RBCs group, which was nanofill. However, the particle sizes for the T material filler system range from 0.11 μm to 15.5 μm, N material filler particles range between 0.1 and 3.0 μm, the C material filler system ranges from 5 nm to 20 μm, and for the O material filler system, 260 nm.

As such, both smaller and bigger particles are involved with C RBCs compared to other filler size systems in the current study, in which larger-sized fillers usually present higher surface roughness, while the small particles adhere more closely to the resin matrix, giving a smoother surface finish^72^. Also, prior researchers reported that any change in the surface roughness impacts the color stability due to their relationship^61,73^. However, the C RBCs showed better color stability, which might confirm that the resin matrix was the major impact factor. Besides, the composite filler type is different: inorganic fillers are used in T, C, and O RBCs, whereas N RBCs have organic-inorganic fillers. The organic component in N RBCs fillers may be a reason for its increased color change compared to T RBCs, which had more filler load, due to its relative water absorption characteristic, which is further increased by the presence of agglomerated particles^74^.

Another explanation, as mentioned by Maghaireh et al.^75^ that the ivocerin photoinitiator system in T RBCs was more absorbent within the violet spectrum than CQ, which was identified in the degree of conversion found with RBCs. More particularly, ivocerin is totally absorbed within the top one to two millimeters of the composite while limiting the transmittance of lower wavelengths responsible for curing in deeper layers. This may lead to incomplete curing at the subsurface layer of RBC, probably affecting the performance and durability of the material. That confirmed by El-Sharawy et al.^76^ which might be another reason that T RBCs have less color stability than other RBCs in the present study.

In the current investigation, the changes in hue across the red-green axis (Da) and the yellow-blue axis (Db) showed that smoking and aging with thermocycling (M3-M1) led to a more reddish and yellowish color in all materials. Staining in subgroup II caused the maximum reddish/yellowish color change, while the least was immersion in saliva. That explains the positive correlation between Da with Db, also Da and Db with DE2000, while DL lowest values in subgroup II, which describes the negative correlation between DL with DE2000 and DL with Da and Db. The lowest DE2000 values were in artificial saliva, and it was noted that immersion in saliva gave positive DL values, indicating that the specimens became lighter. Again, this may be due to the nature of saliva, which is devoid of pigments, and its diluting effect^60^. Also, a positive Db value indicating a slight increase in yellow content might be due to mucin in the composition^17^.

Based on the results of surface roughness in this research, the second null hypothesis was accepted. The results of surface roughness was not significant after exposure (M2), that agreed with Zhao X et al.^15^ who reported that the surface roughness of RBCs was not affected by smoking. On the other hand, it diverges with Alandia-Roman CC et al.^14^ which concluded that smoking impacts the surface roughness of RBCs. These disagreements might be due to the absence of polishing to the RBCs, which influences the outcomes of the RBCs^77^. The results of surface roughness disagree with Botros S et al.^78^ that might be the machine used in their study, as well as different RBCs and exposure products.

Also, the effect of thermocycling (M3) on surface roughness was not significant, and that agreed with El-Rashidy A et al.^52^, and Tuncer S et al.^50^ who reported that thermocycling did not affect the surface roughness of RBCs. Further, disagree with Elmarsafy S et al.^79^ who concluded that thermocycling increased the surface roughness. This disagreement might be due to using different RBCs materials or different finishing and polishing systems. Moreover, from the results of the brushing of RBCs in the current study, there was no significant difference in the surface roughness between the evaluated RBCs. It was in accordance with Karawi M.^80^ while contradicted by Lai G et al.^73^ who reported that the surface roughness of RBCs increased by brushing. This disagreement might be due to using different toothpaste materials with higher RDA than those used in the current research with different RBCs materials.

The relationship between surface roughness and color change in dental RBCs is complex, with many factors affecting it. Although color change itself may not affect surface roughness,^81^ increased in surface roughness could cause or result in a higher degree of color change^82^. The significant influencing factor of surface roughness on the lightness (L*) of RBCs has been pointed out, showing a relation of higher lightness to a decrease in roughness. It is not so easy, however, to single out the surface roughness in relation to chroma (C*) or hue (h*)^83^. Besides, finishing and polishing methods are capable of modifying both surface roughness and color stability, which implies that suitable finishing and polishing procedures will be necessary to maintain the esthetic appearance of restorations^84^. In summary, color change by itself does not have a direct effect on surface roughness, but an increased surface roughness will heighten color changes in dental RBCs. This explanation was confirmed by Elwassefy N et al.^85^.

The low standard deviation values and minimal variation in color change among specimens from the same material group suggest consistent smoke exposure and uniform staining effects across all samples, even though they were placed in different locations within the specimen holder. This reproducibility demonstrates the precision of the experimental design, in which controlled aerodynamics ensured a consistent distribution of smoke particles throughout exposure. These findings confirm the reliability of the exposure apparatus and corroborate earlier research showing the system’s accuracy in mimicking standardized smoking conditions^16^. While the automated processes of brushing, finishing, and polishing by the robot were very repeatable, small standard deviations can be seen within each material with baseline (M1). Nevertheless, minor differences may be attributed to the test material used-filler load, form of filler, and resin matrix. The high loads of fillers may give rougher surfaces, and the filler particles with irregular shapes give a coarser surface texture. The shape of the filler particles alone will affect the final texture; harder resins tend to yield a more irregular finish. These material properties create minor discrepancies in surface texture,^49,86^ because the finishing and polishing process by the robot was very highly precise.

### Limitations

This study has limitations due to its in vitro design, which does not completely replicate the complexity of in vivo conditions, including enzymatic activity, microbial colonization, pH changes, and individual saliva composition variations. Also, the smoking machine method cannot simulate real smoking behavior, individual practices, social environments, and puffing dynamics. Furthermore, this study examined only a limited number of smoke sources and RBCs materials. The study also focuses on color stability and surface roughness, neglecting other properties like mechanical strength and wear resistance. So, the study needs further investigations under clinical conditions.

## Conclusions

Within the limitations of the current study, the following conclusions could be drawn:

- C-cigarettes caused a higher grade of discoloration compared to e-cigarettes, although both types of exposure led to unacceptable discoloration of RBCs. On the other hand, thermocycling, while also contributing to discoloration, had a lesser impact compared to smoking exposure.
- Multi-shade RBCs were more prone to staining and showed lower color stability compared to single-shade RBCs, indicating the resin matrix impact on esthetic performance.
- DL demonstrates a strong negative relationship with Da, Db, and DE2000, on the other hand, positive correlations among Da, Db, and DE2000.
- Neither c-cigarettes nor e-cigarettes exposure, as well as thermocycling, showed a negative effect on surface roughness of RBCs.
- The color change does not directly impact surface roughness, but the increase in roughness can enhance color changes, particularly affecting the light reflection from the materials, which was shown a strong negative relationship with color change.
- The response to exposure relied on the material; not all the materials reacted similarly under the same smoking conditions.
- While offering advanced features, the exposure machine appears to be the standard and controlled means of assessing the effects of smoke on restorative materials. Also, the robot for brushing, finishing, and polishing has shown a precise process.

## Data Availability

The data that support the findings of this study are available from the corresponding author, upon reasonable request.

## References

[1] Barve, D. et al. Assessment of microhardness and color stability of micro-hybrid and nano-filled composite resins. *Niger J. Clin. Pract.* **24**, 1499–1505 (2021). 34657016 10.4103/njcp.njcp_632_20

[2] Kumari, R. V., Nagaraj, H., Siddaraju, K. & Poluri, R. K. Evaluation of the effect of surface polishing, oral beverages and food colorants on color stability and surface roughness of nanocomposite resins. *J. Int. Oral Health*. **7**, 63–70 (2015). 26229373 PMC4513779

[3] Devlukia, S., Hammond, L. & Malik, K. Is surface roughness of direct resin composite restorations material and polisher-dependent? A systematic review. *J. Esthet Restor. Dent.* **35**, 947–967 (2023). 37458370 10.1111/jerd.13102

[4] Cruz Da Silva, E. T. et al. Evaluation of single-shade composite resin color matching on extracted human teeth. *Sci. World J.* **2023**, 1–7 (2023). 10.1155/2023/4376545 PMC10317581 37404241

[5] Iyer, R. S., Babani, V. R., Yaman, P. & Dennison, J. Color match using instrumental and visual methods for single, group, and multi-shade composite resins. *J. Esthet Restor. Dent.* **33**, 394–400 (2021). 32844567 10.1111/jerd.12621

[6] Zhao, X. et al. Effects of different discoloration challenges and whitening treatments on dental hard tissues and composite resin restorations. *J. Dent.* **89**, 103182–103191 (2019). 31430508 10.1016/j.jdent.2019.103182

[7] Paolone, G. et al. In vitro procedures for color stability evaluation of dental resin-based composites exposed to smoke: A scoping review. *Dent. Mater. J.* **41**, 791–799 (2022). 36070929 10.4012/dmj.2022-106

[8] Felicione, N., Karaoghlanian, N., Shihadeh, A., Eissenberg, T. & Blank, M. Comparison of methods of measuring electronic cigarette puff topography. *Tob. Regul. Sci.* **5**, 318–330 (2020). 10.18001/TRS.6.5.2 PMC8096180 33959673

[9] Hmood, A. N., Ebaya, M. M. & El-Embaby, A. E. S. Impact of electronic and conventional cigarettes on color stability and surface roughness of resin-based composite restorative materials: review Article. *Nanotechnol Percept.* **20**, 2815–2829 (2024).

[10] Aydın, N., Topçu, F. T., Karaoğlanoğlu, S., Oktay, E. A. & Erdemir, U. Effect of finishing and Polishing systems on the surface roughness and color change of composite resins. *J. Clin. Exp. Dent.* **13**, 446–454 (2021). 10.4317/jced.58011 PMC8106933 33981391

[11] Elnahas, A. E., Elawsya, M. E. & ElEmbaby, A. E. Impact of different Polishing techniques on surface roughness, gloss, and microhardness of zirconium oxide reinforced flowable bulk-fill resin composite: an in vitro study. *BMC Oral Health*. **25**, 1–14 (2025). 40713603 10.1186/s12903-025-06605-y PMC12297648

[12] Vohra, F. et al. Influence of electronic nicotine delivery systems (ENDS) in comparison to conventional cigarette on color stability of dental restorative materials. *Pak J. Med. Sci.* **36**, 993–998 (2020). 32704277 10.12669/pjms.36.5.2303 PMC7372672

[13] Paolone, G. et al. Color stability of resin-based composites exposed to smoke. A systematic review. *J. Esthet Restor. Dent.* **35**, 309–321 (2023). 36602255 10.1111/jerd.13009

[14] Alandia-Roman, C. C., Cruvinel, D. R., Sousa, A. B. S., Pires-De-Souza, F. C. P. & Panzeri, H. Effect of cigarette smoke on color stability and surface roughness of dental composites. *J. Dent.* **41**, 73–79 (2013). 10.1016/j.jdent.2012.12.004 23270748

[15] Zhao, X. et al. Effects of cigarette smoking on color stability of dental resin composites. *Am. J. Dent.* **30**, 316–322 (2017). 29251454

[16] Hmood, A. N., Ebaya, M. M. A. & El-Embaby, A. E. S. Development of a smoking simulation machine to evaluate the effects of smoking on the color change of dental restorative materials. *Sci. Rep.* **15**, 1–14 (2025). 40295543 10.1038/s41598-025-96898-4 PMC12037891

[17] Ebaya, M. M., Ali, A. I., El-Haliem, H. A. & Mahmoud, S. H. Color stability and surface roughness of ormocer- versus methacrylate-based single shade composite in anterior restoration. *BMC Oral Health*. **22**, 430–442 (2022). 36167560 10.1186/s12903-022-02423-8 PMC9513900

[18] Shamsoddin, E. Dental Floss as an adjuvant of the toothbrush helps gingival health. *EBD* **23**, 94–96 (2022). 36151277 10.1038/s41432-022-0818-x

[19] Ghavami-Lahiji, M. et al. The effect of thermocycling on the degree of conversion and mechanical properties of a microhybrid dental resin composite. *RDE* **43**, 26–38 (2018). 10.5395/rde.2018.43.e26 PMC5952063 29765905

[20] Marigo, L. et al. Influences of different air-inhibition coatings on monomer release, microhardness, and color stability of two composite materials. *Biomed. Res. Int.* **2019**, 1–8 (2019). 10.1155/2019/4240264 PMC6532316 31211136

[21] Elembaby, A. E. S. The effects of mouth rinses on the color stability of resin-based restorative materials. *J. Esthet Dent.* **26**, 264–271 (2014). 10.1111/jerd.12061 24980479

[22] Molaei, M., Mohammadzadeh, A., Ghasemi, A. & Badiee, M. Effect of dry and wet finishing and Polishing on color change and opacity of nanofill and nanohybrid composites. *BMC Oral Health*. **24**, 1–5 (2024). 38419033 10.1186/s12903-024-03944-0 PMC10903010

[23] Heintze, S. D., Reinhardt, M., Müller, F. & Peschke, A. Press-on force during Polishing of resin composite restorations. *Dent. Mater.* **35**, 937–944 (2019). 31005330 10.1016/j.dental.2019.03.009

[24] Alharbi, G., Al Nahedh, H. N. A., Al-Saud, L. M., Shono, N. & Maawadh, A. Effect of different finishing and Polishing systems on surface properties of universal single shade resin-based composites. *BMC Oral Health*. **24**, 1–15 (2024). 38326838 10.1186/s12903-024-03958-8 PMC10848531

[25] Abzal, M. S. et al. Evaluation of surface roughness of three different composite resins with three different Polishing systems. *J. Conserv. Dent.* **19**, 171–174 (2016). 27099426 10.4103/0972-0707.178703 PMC4815548

[26] Tepe, H., Erdilek, A. D., Sahin, M., Efes, B. G. & Yaman, B. C. Effect of different Polishing systems and speeds on the surface roughness of resin composites. *J. Conserv. Dent.* **26**, 36–41 (2023). 36908727 10.4103/jcd.jcd_395_22 PMC10003290

[27] Prochaska, J. J., Vogel, E. A. & Benowitz, N. Nicotine delivery and cigarette equivalents from vaping a JUULpod. *Tob. Control*. **31**, 88–93 (2022). 33762429 10.1136/tobaccocontrol-2020-056367 PMC8460696

[28] Chang, J. T., Anic, G. M., Rostron, B. L., Tanwar, M. & Chang, C. M. Cigarette smoking reduction and health risks: a systematic review and meta-analysis. *N&TR* **23**, 635–642 (2021). 10.1093/ntr/ntaa156 32803250

[29] Alebady, M. H., Hamama, H. H. & Mahmoud, S. H. Effect of various surface coating methods on surface roughness, micromorphological analysis and fluoride release from contemporary glass ionomer restorations. *BMC Oral Health*. **24**, 1–15 (2024). 38685036 10.1186/s12903-024-04234-5 PMC11057179

[30] Lorenz, J. et al. In vitro surface analysis of the brushing resistance of orthodontic sealants using two different profilometric evaluation methods. *Sci. Rep.* **12**, 1–9 (2022). 36167702 10.1038/s41598-022-19702-7 PMC9515092

[31] Singh, G., Agarwal, A. & Lahori, M. Effect of cigarette smoke on the surface roughness of two different denture base materials: an in vitro study. *J. Indian Prosthodont. Soc.* **19**, 42–48 (2019). 30745753 10.4103/jips.jips_82_18 PMC6340078

[32] Hamdy, T. M., Abdelnabi, A., Othman, M. S., Bayoumi, R. E. & Abdelraouf, R. M. Effect of different mouthwashes on the surface microhardness and color stability of dental nanohybrid resin composite. *J. Polym.* **15**, 815–826 (2023). 10.3390/polym15040815 PMC9961015 36850099

[33] Alhotan, A. et al. Colour parameters and changes of tea-stained resin composite exposed to whitening pen (in vitro study). *J. Polym.* **15**, 3068–3080 (2023). 10.3390/polym15143068 PMC10383341 37514457

[34] Yerliyurt, K. & Sarıkaya, I. Color stability of hybrid ceramics exposed to beverages in different combinations. *BMC Oral Health*. **22**, 180–192 (2022). 35568863 10.1186/s12903-022-02206-1 PMC9107770

[35] Rohanová, D., Horkavcová, D., Helebrant, A. & Boccaccini, A. R. Assessment of in vitro testing approaches for bioactive inorganic materials. *J. Non Cryst. Solids*. **432**, 53–59 (2016).

[36] Paolone, G., Diana, C. & Cantatore, G. State-of-the-art in resin-based composites and future trends. *Compend Contin Educ Dent* **44**, 98–100 (2023). 36802751

[37] Maran, B. M. et al. Nanofilled/nanohybrid and hybrid resin-based composite in patients with direct restorations in posterior teeth: A systematic review and meta-analysis. *J. Dent.* **99**, 103407 (2020). 32526348 10.1016/j.jdent.2020.103407

[38] Cruz Da Silva, E. T. et al. Evaluation of single-shade composite resin color matching on extracted human teeth. *Sci. World J.* **2023**, 4376545–4376553 (2023). 10.1155/2023/4376545 PMC10317581 37404241

[39] Zhu, J., Xu, Y., Li, M. & Huang, C. Instrumental and visual evaluation of the color adjustment potential of a recently introduced single-shade composite resin versus multishade composite resins. *J. Prosthet. Dent.* **134**, 832–839. 10.1016/j.prosdent.2023.09.037 (2023). 10.1016/j.prosdent.2023.09.037 37919131

[40] Vallone, D. M. et al. Electronic cigarette and Juul use among adolescents and young adults. *JAMA Pediatr.* **174**, 277–286 (2020). 31961395 10.1001/jamapediatrics.2019.5436 PMC6990671

[41] Stone, M. D. et al. Effects of cigarette package colors and warning labels on marlboro smokers’ risk beliefs, product appraisals, and smoking behavior: a randomized trial. *BMC Public. Health*. **23**, 1–12 (2023). 37891513 10.1186/s12889-023-17024-5 PMC10605973

[42] Can Say, E., Yurdagüven, H., Yaman, B. C. & Özer, F. Surface roughness and morphology of resin composites polished with two-step Polishing systems. *Dent. Mater. J.* **33**, 332–342 (2014). 24598241 10.4012/dmj.2013-287

[43] Pires, P. M. et al. Bonding performance and interfacial adaptation of modern bulk-fill restorative composites after aging in artificial saliva: an in vitro study. *Clin. Oral Investig*. **28**, 1–16 (2024). 10.1007/s00784-024-05525-5 38308668

[44] Labib, M. E. et al. Stain removal, abrasion and anticaries properties of a novel low abrasion dentifrice containing micro-fibrillated cellulose: in vitro assessments. *J. Dent.* **146**, 105038 (2024). 38714242 10.1016/j.jdent.2024.105038

[45] Paolone, G. et al. Color stability of resin-based composites: staining procedures with liquids - a narrative review. *J. Esthet Restor. Dent.* **34**, 865–887 (2022). 35396818 10.1111/jerd.12912

[46] Alnasser, H. A., Elhejazi, A. A., Al-Abdulaziz, A. A., Alajlan, S. S. & Habib, S. R. Effect of conventional and electronic cigarettes smoking on the color stability and translucency of tooth colored restorative materials: an in vitro analysis. *Coatings* **11**, 1568–1577 (2021).

[47] Abdulla, R. & Cowan, K. Smoke and mirrors – does smoking cause discolouration of composite restorations? *Evid. Based Dent.* **24**, 157–158 (2023). 37993688 10.1038/s41432-023-00955-8 PMC10724062

[48] Gömleksiz, S. & Okumuş, Ö. F. The effect of whitening toothpastes on the color stability and surface roughness of stained resin composite. *BMC Oral Health*. **24**, 1–9 (2024). 39069637 10.1186/s12903-024-04654-3 PMC11283717

[49] Naji, W. D. & Abd-Alla, M. H. Effect of reduced-step Polishing systems on color stability of nanocomposites submitted to thermocycling staining with common beverages - an in vitro study. *Indian J. Dent. Res.* **35**, 191–195 (2024). 39282760 10.4103/ijdr.ijdr_182_23

[50] Tuncer, S., Demirci, M., Tiryaki, M., Ünlü, N. & Uysal, Ö. The effect of a modeling resin and thermocycling on the surface hardness, roughness, and color of different resin composites. *J. Esthet Restor. Dent.* **25**, 404–419 (2013). 24172016 10.1111/jerd.12063

[51] Hajj, R. J., Nasr, L., Khairallah, C. & Hardan, L. In vitro assessment of the color stability of two resin composites. *Curr. Res. Dent.* **14**, 30–40 (2023).

[52] El-Rashidy, A. A., Shaalan, O., Abdelraouf, R. M. & Habib, N. A. Effect of immersion and thermocycling in different beverages on the surface roughness of single- and multi-shade resin composites. *BMC Oral Health*. **23**, 1–8 (2023). 37287027 10.1186/s12903-023-03069-w PMC10249292

[53] Mathias, P., Santos, S. R., Aguiar, T. R., Santos, P. R. & Cavalcanti, A. N. Cigarette smoke: effects on water sorption and solubility of restorative dental composites. *Gen. Dent.* **62**, 54–57 (2014). 24598497

[54] Gusmão, G. M., De Queiroz, T. V., Pompeu, G. F., Menezes Filho, P. F. & da Silva, C. H. The influence of storage time and pH variation on water sorption by different composite resins. *Indian J. Dent. Res.* **24**, 60–65 (2013). 23852234 10.4103/0970-9290.114954

[55] Bociong, K. et al. The influence of water sorption of dental light-cured composites on shrinkage stress. *Mater. (Basel)*. **10**, 1–14 (2017). 10.3390/ma10101142 PMC5666948 28956844

[56] Huang, W. et al. Evaluation of the color stability, water sorption, and solubility of current resin composites. *Mater. (Basel)*. **15**, 1–14 (2022). 10.3390/ma15196710 PMC9572228 36234048

[57] Biçer, Z., Yaman, B. C., Çeliksöz, Ö. & Tepe, H. Evaluation of the color stability of resin composites subjected to artificial aging procedures: immersion in different solutions, brushing, and thermal cycling. *Odontology* **113**, 1003–1020. 10.1007/s10266-024-01038-5 (2025). 10.1007/s10266-024-01038-5 39693034

[58] Koc-Vural, U., Baltacioglu, I. & Altinci, P. Color stability of bulk-fill and incremental-fill resin-based composites polished with aluminum-oxide impregnated disks. *RDE* **42**, 118–124 (2017). 28503477 10.5395/rde.2017.42.2.118 PMC5426223

[59] Porojan, L., Toma, F. R., Uțu, I. D. & Vasiliu, R. D. Optical behavior and surface analysis of dental resin matrix ceramics related to thermocycling and finishing. *Appl. Sci.* **12**, 4346 (2022).

[60] El-Rashidy, A. A., Abdelraouf, R. M. & Habib, N. A. Effect of two artificial aging protocols on color and gloss of single-shade versus multi-shade resin composites. *BMC Oral Health*. **22**, 321–333 (2022). 35915423 10.1186/s12903-022-02351-7 PMC9341039

[61] Alex, A. & Venkatesh, V. Comparative evaluation of surface roughness and color stability between single-shade composite and multi-shade composite: an in vitro study. *Cureus* **16**, 1–10 (2024). 10.7759/cureus.65396 PMC11344600 39184644

[62] Khayat, W. F. In vitro comparison of optical properties between single-shade and conventional composite resin restorations. *Cureus* **16**, 57664–57573 (2024). 10.7759/cureus.57664 PMC11070174 38707099

[63] Alalawi, H. Evaluation of staining, color stability, blending effect, and depth of cure of a new universal resin composite [Nova Southeastern University ProQuest Dissertations & Theses]. [Florida, United States]: 30312032; (2023).

[64] Maghaireh, G., Alzraikat, H. & Alakhras, H. Surface roughness and color stability of newly developed resin composites with color adjustment potential after immersion in staining solutions. *Oper. Dent.* **1**, 67–77 (2025). 10.2341/24-047-L 39601698

[65] Duzyol, M., Duzyol, E. & Çarıkçıoğlu, B. Assessing one-shade composite resin color stability in response to everyday drinks. *BMC Oral Health*. **24**, 1–8 (2024). 39033129 10.1186/s12903-024-04612-z PMC11264926

[66] Priya, B., Arora, A. & Taneja, S. Spectrophotometric evaluation of color stability of novel composites following exposure to antioxidant beverages: an in vitro study. *JCDE* **27**, 866–872 (2024). 39372579 10.4103/JCDE.JCDE_55_24 PMC11451687

[67] Soliman, Y. A., Mahmoud, E. M., Gepreel, M. H. & Afifi, R. R. The ability of coffee to stain nanohybrid composite resins. *ADJALEXU* **46**, 91–95 (2021).

[68] Sensi, L., Winkler, C. & Geraldeli, S. Accelerated aging effects on color stability of potentially color adjusting resin-based composites. *Oper. Dent.* **46**, 188–196 (2021). 34086953 10.2341/20-099-L

[69] Meena, K. et al. Color stability of posterior nanocomposites treated with colored beverages after brushing and thermocycling: an in vitro study. *Int. J. Oral Health Sci.* **12**, 370–377 (2020).

[70] Barutcigil, Ç., Barutcigil, K., Özarslan, M. M., Dündar, A. & Yilmaz, B. Color of bulk-fill composite resin restorative materials. *J. Esthet Restor. Dent.* **30**, 3–8 (2018). 10.1111/jerd.12340 28960790

[71] Pala, K., Tekçe, N., Tuncer, S., Serim, M. E. & Demirci, M. Evaluation of the surface hardness, roughness, gloss and color of composites after different finishing/polishing treatments and thermocycling using a multitechnique approach. *Dent. Mater. J.* **35**, 278–289 (2016). 27041019 10.4012/dmj.2015-260

[72] Khairy, A. A., El-Toukhy, R. I. & Zaghlol, N. Effect of finishing/polishing techniques on surface roughness of three different resin composite materials: a laboratory study. *MJD* **9**, 82–88 (2022).

[73] Lai, G., Zhao, L., Wang, J. & Kunzelmann, K. H. Surface properties and color stability of dental flowable composites influenced by simulated toothbrushing. *Dent. Mater. J.* **37**, 717–724 (2018). 29998939 10.4012/dmj.2017-233

[74] ÇOBANOĞLU, N., GÜNGÖR, F., Abdulateef, O. F. & VELİOĞLU, M. S. Effect of translucency on color stability of resin-based composites. *Bezmialem Sci.* **11**, 213–220 (2023).

[75] Maghaireh, G. A., Price, R. B., Abdo, N., Taha, N. A. & Alzraikat, H. Effect of thickness on light transmission and Vickers hardness of five bulk-fill resin-based composites using Polywave and single-peak light-emitting diode curing lights. *Oper. Dent.* **44**, 96–107 (2019). 29953339 10.2341/17-163-L

[76] El-Sharawy, R. M., Elawsya, M. E., Abdallah, A. M. & Elembaby, A. E. Depth of cure of bulk-fill resin composites with different photo-initiator systems cured by monowave and Polywave light curing units. *Egypt. Dent. J.* **70**, 2083–2091 (2024).

[77] Kobayashi, M. et al. Isolated effect of filler particle size on surface properties of experimental resin composites before and after toothbrush abrasion. *J. Esthet Restor. Dent.* **35**, 1286–1292 (2023). 37449726 10.1111/jerd.13105

[78] Botros, S. A. et al. Effect of smoking and finishing/polishing on roughness of nanohybrid-composite. *Int. Dent. J.* **74**, 306 (2024).

[79] Elmarsafy, S. M., Abdelwahab, S. A. & Hussein, F. Influence of Polishing systems on surface roughness of four resin composites subjected to thermocycling aging. *J. Dent. Res.* **20**, 1–9 (2023). PMC10166756 37180693

[80] Karawi, M. Evaluation of physical properties of a new universal resin composite with emphasis on surface characteristics and radiopacity [Nova Southeastern University ProQuest Dissertations & Theses]. [Florida, United States]: 28860905; (2021).

[81] Hajdu, A. I. et al. Enhancing esthetics in direct dental resin composite: investigating surface roughness and color stability. *J. Funct. Biomater.* **15**, 208–223 (2024). 39194646 10.3390/jfb15080208 PMC11355370

[82] Alonazi, M. Impact of smoking on resin bonded restorations: A narrative review. *Tob. Induc. Dis.* **22**, 1–9 (2024). 10.18332/tid/188114 PMC11135022 38813584

[83] Ghinea, R. et al. Influence of surface roughness on the color of dental-resin composites. *J. Zhejiang Univ. Sci. B*. **12**, 552–562 (2011). 21726062 10.1631/jzus.B1000374 PMC3134843

[84] Yildiz, E., Sirin Karaarslan, E., Simsek, M., Ozsevik, A. S. & Usumez, A. Color stability and surface roughness of polished anterior restorative materials. *Dent. Mater. J.* **34**, 629–639 (2015). 25925685 10.4012/dmj.2014-344

[85] Elwassefy, N., ElEmbaby, A. & Elkholany, N. Correlation between surface roughness and color stability of nano- and micro-hybrid resin composites using different surface treatment protocols. *Egypt. Dent. J.* **69**, 705–713 (2023).

[86] Alali, M., Silikas, N. & Satterthwaite, J. The effects of toothbrush wear on the surface roughness and gloss of resin composites with various types of matrices. *Dent. J. (Basel)*. **9**, 1–11 (2021). 10.3390/dj9010008 PMC7827053 33445457

